# A case report of reversible cerebral vasoconstriction syndrome with thunderclap headache significantly exacerbated in the supine position and alleviated in the standing position

**DOI:** 10.1186/s12883-023-03381-6

**Published:** 2023-10-03

**Authors:** Genri Toyama, Shintaro Tsuboguchi, Kazuya Igarashi, Etsuji Saji, Takuya Konno, Osamu Onodera

**Affiliations:** Department of Neurology, Niigata University, 1-757 Asahimachidori, Chuo-ku, Niigata city, 951-8585 Niigata Japan

**Keywords:** Reversible cerebral vasoconstriction syndrome, Thunderclap headache, Intracranial pressure, Postpartum period.

## Abstract

**Background:**

Reversible cerebral vasoconstriction syndrome (RCVS) is characterized by sudden onset thunderclap headache and multiple segmental reversible cerebral vasoconstrictions that improve within 3 months. The postpartum period is a well-known precipitating factor for the onset of RCVS. Cerebral venous thrombosis (CVT) causes thunderclap headaches in the postpartum period. While headache in CVT is sometimes exacerbated in the supine position, the severity of the headache in RCVS is usually independent of body position. In this study, we report a case of RCVS with thunderclap headache exacerbated in the supine position, and headache attacks that resolved quickly in the standing position during the postpartum period.

**Case presentation:**

A 33-year-old woman presented with a sudden increase in blood pressure and thunderclap headache on the fifth postpartum day (day 1: the first sick day). The headache was severe and pulsatile, with onset in the supine position in bed, and peaked at approximately 10 s. It was accompanied by nausea and chills but there were no scintillating scotomas or ophthalmic symptoms. The headache resolved in the standing or sitting position but was exacerbated and became unbearable within a few seconds when the patient was in the supine position. Therefore, she was unable to lie supine at night. Computed tomography angiography (CTA) of the head on day 2 and magnetic resonance imaging (MRI) and magnetic resonance angiography (MRA) on day 3 showed no abnormalities. However, considering the possibility of RCVS, verapamil was initiated on day 3. The headache resolved the following day. MRA of the head on day 10 revealed diffuse and segmental stenoses in the bilateral middle and posterior cerebral arteries and basilar artery. Therefore, the patient was diagnosed with RCVS. The headache gradually resolved and disappeared completely on day 42. Cerebral vasoconstriction was also improved on MRA on day 43.

**Conclusions:**

This postpartum RCVS case was notable for the exacerbation of headaches in the supine position. For the diagnosis of thunderclap headache in the postpartum period, RCVS should be considered in addition to CVT when the patient presents with a headache that is exacerbated in the supine position.

## Background

Reversible cerebral vasoconstriction syndrome (RCVS) is a disorder associated with sudden onset thunderclap headache and multiple segmental reversible cerebral vasoconstrictions that improve within three months [1]. Sympathetic nervous system hyperactivity causes cerebral vascular dysregulation, which progresses from the peripheral small arteries to the middle and large arteries [1]. Cerebral perfusion is reduced owing to stenosis of the distal cerebral arteries [2]. Hypoperfusion causes leptomeningeal artery hyperdilation and causes headache [3–5]. The headache is paroxysmal, peaking within a few minutes, lasting from 5 min to 36 h (average, 5 h), and is thunderclap-like [6].

RCVS and other diseases such as idiopathic intracranial hypertension, eclampsia, cerebral venous thrombosis (CVT), pituitary apoplexy, and post-dural puncture can cause postpartum headaches [7]. Among these, CVT can present with a thunderclap headache. The nature of the headache is more suggestive of CVT when exacerbated in the supine position [7]. However, the severity of headaches in RCVS is usually independent of body position.

Here, we report a case of postpartum RCVS with a thunderclap headache that was exacerbated in the supine position and quickly resolved in the standing position. This case suggests the differential diagnosis of thunderclap headache during the postpartum period, which was exacerbated in the supine position.

## Case presentation

A 33-year-old woman with no medical history was admitted to the obstetrics department because of pregnancy with uterine fibroids. She delivered naturally without anesthesia. This was the patient’s first delivery. She had no hypertension during pregnancy, but the systolic blood pressure sometimes exceeded 150 mmHg from the fourth postpartum day onward. She was discharged on the fifth postpartum day (the first day of sickness) with a morning blood pressure of 158/82 mmHg. Upon returning home, the patient experienced a mild headache. At approximately 22:00 that night, 10 min after getting into bed, she suddenly felt chills and had a severe, pulsating headache that peaked in approximately 10 s. Nausea was accompanied by headache, but there were no scintillating scotomas or ophthalmic symptoms. Her blood pressure was 159/100 mmHg. The headache resolved in the standing position but worsened and became unbearable within a few seconds when she was in the supine position. Therefore, she was unable to lie down all night. The following day, the patient visited our hospital. Her blood pressure was 156/110 mmHg at the time of examination. Acetaminophen and diazepam did not improve the headache, and she was admitted on the same day.

**Fig. 1:**
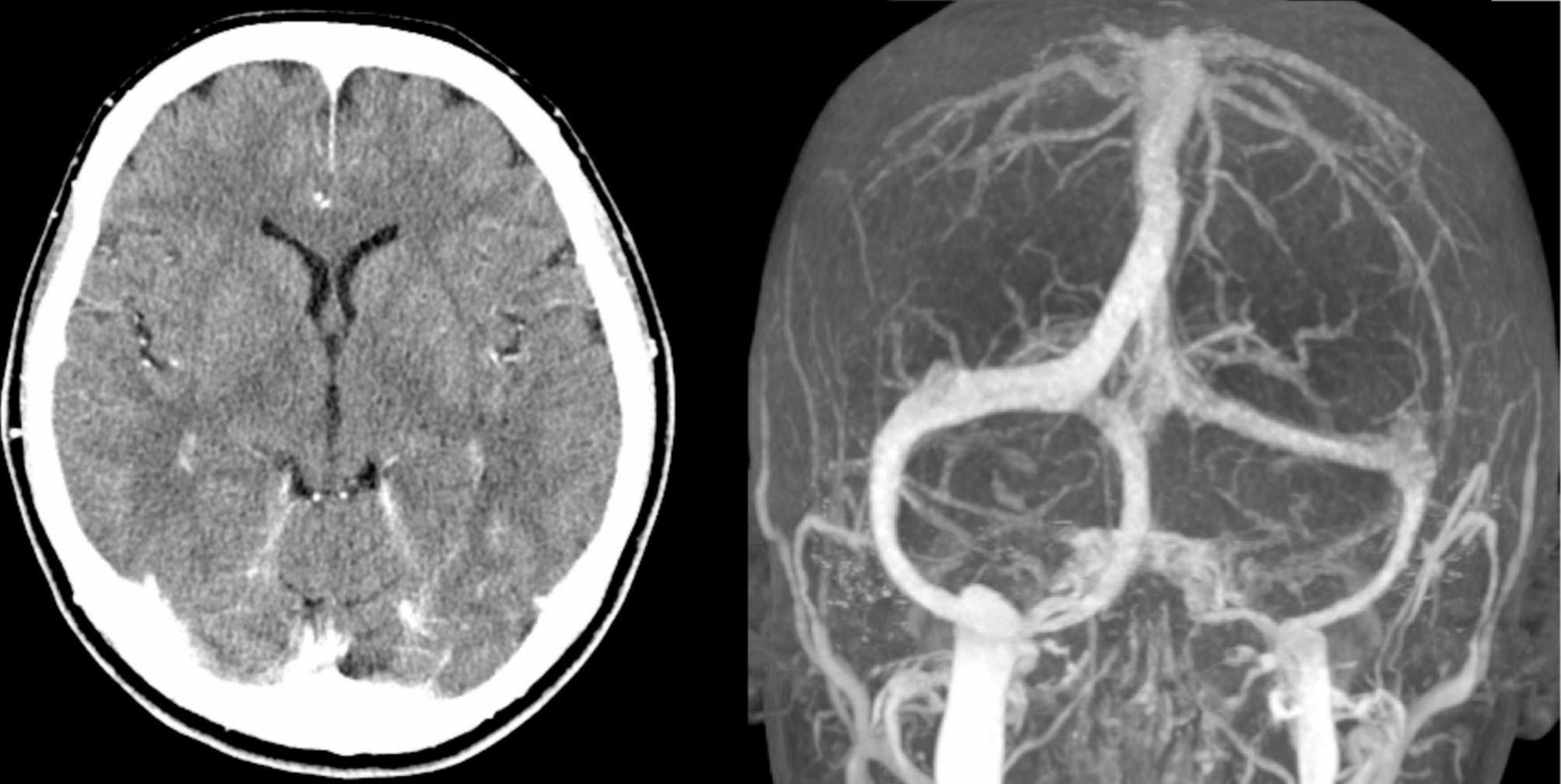
Computed tomography and computed tomography venography in the case. There were no abnormalities in the brain parenchyma, and no venous occlusion

On admission, her body temperature was 35.6 °C, and her blood pressure was 178/110 mmHg. No abnormalities were found upon physical examination of the chest or abdomen. Consciousness was clear, and there were no neurological symptoms, including nuchal rigidity. Laboratory findings showed a white blood cell count of 7390/µl, C-reactive protein 0.94 mg/dl, and no anemia. Liver and renal function were normal, and electrolyte levels were within the normal range. D-dimer was elevated at 8.1 µg/ml, but there was no other abnormality in the coagulation system. Contrast-enhanced computed tomography (CT) of the brain on admission (day 2) at the time of headache relief showed no abnormalities, and computed tomography angiography (CTA) showed no vascular stenosis or venous occlusion (Fig. 1).

**Fig. 2:**
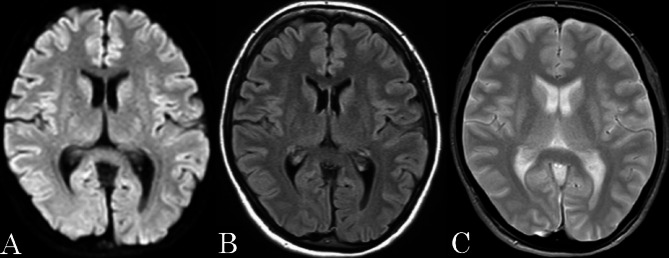
Brain magnetic resonance imaging on the day 3. **A**: Diffusion-weighted image. **B**: Fluid-attenuated Inversion recovery. **C**: T2 star-weighted image. There were no abnormalities in the brain parenchyma, or thrombosed vein sign

**Fig. 3:**
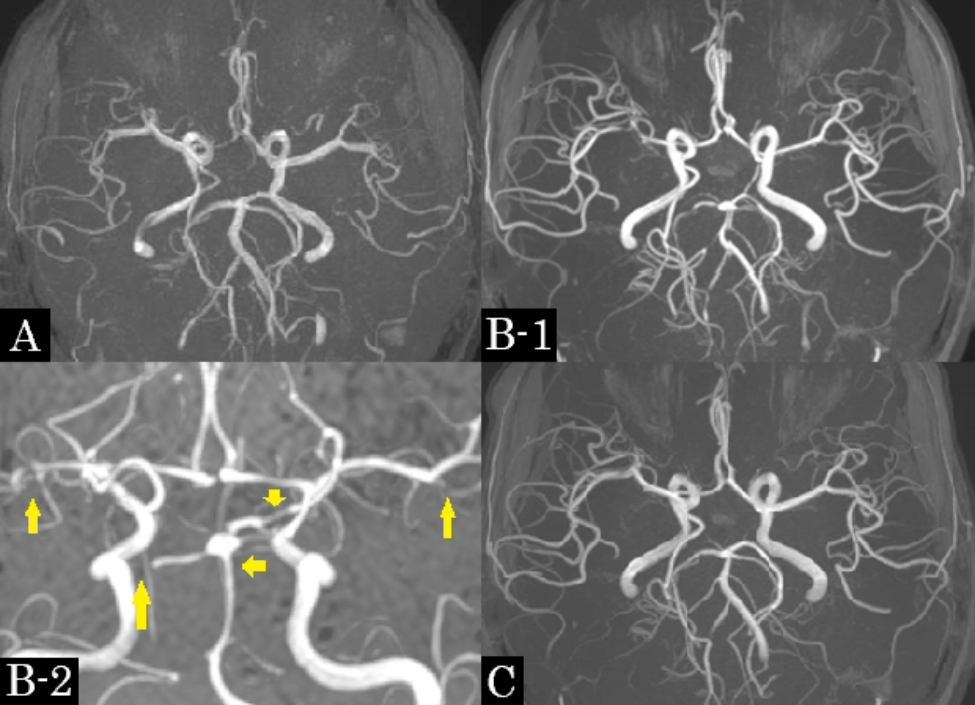
Magnetic resonance angiography. **A**; Day 3. Peripheral blood vessels were poorly revealed compared to later images. **B**-1; Day 10. **B**-2: Day 10, magnified view of the different angles from B1. Diffuse segmental stenoses in the bilateral middle cerebral arteries, bilateral posterior cerebral arteries, and basilar artery (arrows). **C**; Day 43

After admission, the headache pulsated over the entire head and became extremely severe within 10 s in the supine position. The headache resolved within a few seconds in the standing or sitting position but did not disappear. Headache intensity was variable and exacerbated for approximately 10 min/h. Scintillating scotomas were not observed. One hour after nifedipine administration, the headache decreased, and she was able to sleep in a sitting position for a short time on day 2. On the third day after discharge, brain magnetic resonance imaging (MRI) showed no direct thrombosed vein sign and no other abnormalities (Fig. 2), but magnetic resonance angiography (MRA) showed poor visualization of peripheral arteries (Fig. 3A).　We suspected RCVS and prescribed verapamil 120 mg/day (in three divided doses). After taking verapamil at night, her headache resolved approximately 1 h later and she was able to lie in a supine position. On day 4, she experienced a thunderclap headache preceded by chills when she got to bed; however, she did not experience any subsequent thunderclap headache. On day 10, brain MRA (Fig. 3B) showed diffuse and segmental stenoses in the bilateral middle and posterior cerebral arteries and basilar artery. Thereafter, she experienced a mild headache once a week for several hours; however, the headache did not exacerbate in the supine position. The headaches completely disappeared by day 42. MRA performed on day 43 (Fig. 3C) showed an improvement in vascular stenosis. Verapamil was discontinued on day 62; however, the headache did not relapse.

## Discussion and conclusions

This case was characterized by a thunderclap headache that developed in the supine position, was relieved in the standing position, and exacerbated in the supine position. Secondary headache tends to increase in the last trimester and postpartum period [7]. In the differential diagnosis of secondary headaches, we should consider vascular disease, CVT, pituitary apoplexy, brain neoplasm, post-dural puncture headache, RCVS, and idiopathic intracranial hypertension. In addition, subarachnoid hemorrhage, RCVS, CVT, cervical artery dissection, and posterior reversible encephalopathy (PRES), are known to cause thunderclap headaches [8]. Headaches are exacerbated in the supine position due to intracranial hypertension, such as CVT, brain neoplasm, and hydrocephalus [9, 10]; However, headache in RCVS is not considered to fluctuate with body position. In this case, we considered CVT because the thunderclap headache in the postpartum period and exacerbated in the supine position; however, a diagnosis of RCVS was made based on imaging findings.

Three cases of RCVS with headaches exacerbated in the supine position have been reported (Table 1). The first case involved a 30-year-old woman with a history of systemic lupus erythematosus and migraines, who developed a thunderclap headache on the sixth day after delivery, followed by radiating pain from the frontal region to the neck, mild nausea, photophobia, and phonophobia [11]. The headache persisted, and on day 7, a secondary generalized seizure occurred, and consciousness was disturbed for two days. Cerebrospinal fluid analysis, brain CT, and MRI on day 1 were normal. However, MRI on day 8 showed posterior reversible encephalopathy syndrome, and MRA on day 14 showed multiple segmental cerebral vascular stenoses. Antihypertensive therapy and magnesium sulfate were administered, and the patient’s symptoms improved after two months. The second case was that of a 17-year-old woman who presented with a hammer-striking thunderclap headache and convulsive seizures lasting 3–4 h, followed by disturbance of consciousness, resulting in cerebral infarction [12]. MRA showed multiple transient vasoconstrictions of the basilar artery, resulting in a diagnosis of RCVS. In this case, bilateral subclavian artery occlusions were observed and Takayasu’s arteritis was diagnosed as a complication. The third case was that of a 34-year-old woman who developed a pulsatile thunderclap headache immediately after an argument with her husband on the fourth day after delivery, and even the slightest body movement caused severe headache [13]. MRA on day 6 showed multiple stenoses in the bilateral middle cerebral arteries, anterior cerebral arteries, and posterior cerebral arteries. The headache improved after the administration of verapamil and glycerin. In all three cases, cerebral vascular stenoses eventually improved.

**Table 1:** Cases of reversible cerebral vasoconstriction syndrome with headache exacerbated in the supine position

Authors and year	Lemmens et al. (2012) [11]	Lee et al. (2013) [12]	Gotoh et al. (2019) [13]	This case
Patient	30 y.o. female	17 y.o. female	34 y.o. female	33 y.o. female
Past history	systemic lupus erythematosus, migraine	headache	migraine, anxiety disorder	uterine fibroids
Onset	6th postpartum day	after the seizure	4th postpartum day	5th postpartum day
Headache	thunderclap with nausea, photophobia	thunderclap (Hammer-striking)	thunderclap (body movement exacerbates)	thunderclap with nausea, chills
Blood pressure	129/87 mmHg	154/95 mmHg	113/56 mmHg	158/82 mmHg
CSF pressure	Normal pressure	40 cmH_2_O	Not examined	Not examined
Imaging findings	diffuse hyperintensity in cortex and subcortex on FLAIR multiple stenoses in bilateral MCA and PCA	diffusion restriction in bilateral occipital and frontal lobe multiple stenoses in basilar artery	hyperintense vessel sign on FLAIR multiple stenoses in bilateral MCA and PCA	multiple stenoses in bilateral MCA and PCA and basilar artery
Complication	PRES	cerebral infarction, Takayasu’s arteritis, convulsive seizure	None	None
Treatment	magnesium sulfate, antihypertensive treatment	IVMP 1000 mg 3 times, nimodipine 240 mg/day, oral methylprednisolone	verapamil 120 mg/day, concentrated glycerin	verapamil 120 mg/day
Outcome	symptoms and vascular stenoses improved in 2 months	symptoms and cerebral vascular stenoses improved in 1 month	symptoms improved in 10 days, vascular stenoses improved in 2 months	symptoms and vascular stenoses improved on day 43

CSF, cerebrospinal fluid; FLAIR, Fluid-attenuated inversion recovery; PRES, posterior reversible encephalopathy syndrome; MCA, middle cerebral artery; PCA, posterior cerebral artery; IVMP, intravenous methylprednisolone

Increased sympatheticactivity can cause dysregulation of cerebral vascular tone, which is known to be one of the pathogenetic factors of RCVS [1]. The second case described above was associated with increased intracranial pressure. When the body position changes from standing to supine, increased intracranial pressure can cause increased sympathetic nerve activity [14]. The second and present cases presented with hypertension, which may be related to increased sympathetic nerve activity. It is noteworthy that the first, third and present cases had postpartum onset. It has been reported that when changing from an upright to a supine position, nonpregnant women have higher vagal activity and lower sympathetic activity, whereas pregnant women have lower vagal activity and higher sympathetic activity due to aortocaval compression by the gravid uterus [15]. Since it takes 6–8 weeks for the uterus to return to its pre-pregnancy size after delivery [16], the uterus in the early postpartum period could be large enough to cause aortocaval compression. Therefore, during the early postpartum period, when a woman is in the supine position, her enlarged uterus can compress the aorta and excite the sympathetic nerves. We suspect that this sympathetic stimulation exacerbated the thunderclap headache in this RCVS patient.

**Fig. 4:**
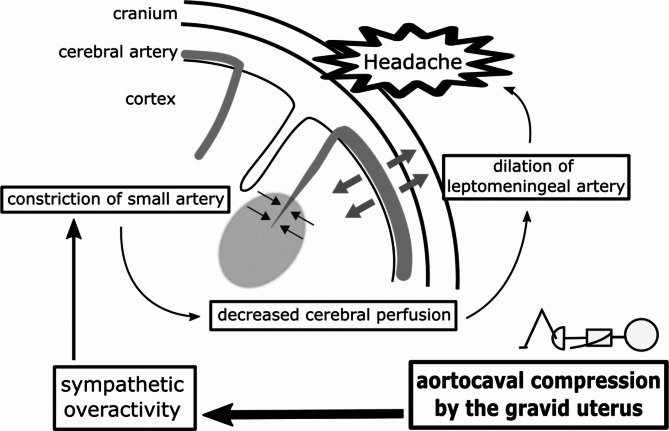
Hypothesis on the exacerbation of headache in the supine position in reversible cerebral vasoconstriction syndrome. In RCVS, cerebral perfusion is decreased by the constriction of the small cerebral arteries. To compensate for the decreased cerebral perfusion, the leptomeningeal arteries are dilated, resulting in severe headaches. In the early postpartum period, higher sympathetic activity due to aortocaval compression by the gravid uterus in the supine position contributes to dysregulation of the small cerebral arteries and exacerbates headaches

The common mechanism of headache, which is exacerbated in the supine position, involves an increase in intracranial pressure. It has been reported that normal human reference values for intracranial pressure were − 5.9 to 8.3 mmHg in the upright position and 0.9 to 16.3 mmHg in the supine position [17]. Therefore, in diseases that cause significant edema of the brain parenchyma, such as CVT, the supine position further increases intracranial pressure and exacerbates headaches. In RCVS, meanwhile, the headache usually does not exacerbate in the supine position, therefore intracranial pressure may increase within the normal range. It has been proposed that stenosis of the small cerebral arteries causes decreased cerebral perfusion, and decreased cerebral perfusion contributes to compensatory hyperdilation of the leptomeningeal arteries [3–5]. In our case, we hypothesized that the supine position increased sympathetic activity due to aortocaval compression by the gravid uterus and promote dysregulation of cerebral vascular tone, resulting in headache exacerbation (Fig. 4). Therefore, if the headache is exacerbated in the supine position, it may be better to avoid the supine position.

This report has some limitations. First, there are few reports of RCVS in which headache is exacerbated in the supine position, and we have not yet found enough commonalities. Second, intracranial pressure was not measured in this case. While the second previously case presented increased intracranial pressure, there is a report of RCVS caused by decreased intracranial pressure [18]. Further studies are required to fully elucidate the relationship between headache and intracranial pressure in RCVS.

When a thunderclap headache is present during the postpartum period and is exacerbated in the supine position, RCVS should be included in the differential diagnosis, in addition to CVT.

## Data Availability

All data related to this case report are documented within this manuscript.

## Abbreviations

RCVS: reversible cerebral vasoconstriction syndrome
CVT: cerebral venous thrombosis
CT: computed tomography
CTA: computed tomography angiography
MRI: magnetic resonance imaging
MRA: magnetic resonance angiography
FLAIR: Fluid-attenuated inversion recovery
PRES: posterior reversible encephalopathy syndrome
MCA: middle cerebral artery
PCA: posterior cerebral artery
IVMP: intravenous methylprednisolone
